# Gastrobronchial Fistula: A Rare Complication of Postlaparascopic Sleeve Gastrectomy—A Case Report and Literature Review

**DOI:** 10.1155/2021/6641319

**Published:** 2021-03-17

**Authors:** Mohammed Sabawi, Alhassan Alhasson, Abdul-Rahman Abualruz, Alaa Abdulsattar Al-Taie

**Affiliations:** ^1^Department of Radiology, Hamad Medical Corporation, Doha, Qatar; ^2^Department of Radiology, Wake Forest University Baptist Medical Center, Medical Center Boulevard, Winston-Salem, NC 27157, USA

## Abstract

**Introduction:**

Obesity is one of the leading causes of morbidity and mortality in countries all over the world, and its prevalence has been increasing dramatically in recent years. Bariatric surgery is considered the gold standard of care for patients who failed conservative management. Laparoscopic sleeve gastrectomy (LSG) is of increasing popularity. One of its vicious consequences is the development of acquired fistula between the stomach and the tracheobronchial tract due to intractable gastric leak. *Case Report*. We are presenting a case of a 25-year-old man who underwent laparoscopic sleeve gastrectomy for morbid obesity, which was complicated with the development of gastrobronchial fistula, despite an unremarkable postoperative course.

**Conclusion:**

Acquired gastrobronchial fistula due to bariatric surgery is not reported widely in radiologic literature; hence, there is lack of consensus of the diagnostic modality of choice. However, there is a myriad of tests available for diagnosing gastrobronchial fistula, with contrast study of the upper gastrointestinal tract which is the widely accepted diagnostic test.

## 1. Introduction

Obesity is one of the leading causes of morbidity and mortality all over the world [1], and its prevalence has been increasing dramatically in recent years [2]. Bariatric surgery is considered the gold standard of care for patients who failed the initial management of lifestyle modification, diet modification, and routine physical activity [3]. While RYGB remains the most common bariatric operation globally, sleeve gastrectomy is gaining huge popularity by surgeons because of its proven efficacy, easier surgical technique, and less adverse effects compared to RYGB [4, 5]. LSG is a restrictive procedure comprises of the laparoscopic resection of the greater curvature of the stomach, including most of the fundus and corpus; weight loss is achieved due to decreased food intake as a result of reduction of gastric volume and fundal gastric cells that produce ghrelin (hunger-inducing hormone) [6].

The most common chronic complications of LSG are gastroesophageal reflux disease and gastric stricture [7], while hemorrhage along the staple line (<5%) and gastric fistula formation (<2%) is the most prevalent acute adverse events [8]. Up to our knowledge post-LSG gastrobronchial fistula (GBF) is rarely reported in radiologic literature. Despite its rarity, it is a paramount complication that needs to be studied due to its difficult diagnosis and management with high mortality and morbidity rate [9]. The management of GBF is challenging with no clear treatment guidelines; however, it requires a multidisciplinary approach, including radiological, endoscopic, and surgical interventions [10]. We are presenting a case of a 25-year-old male who presented with GBF following LSG with aiming to familiarize radiologists specifically and clinicians of its clinical and radiographic features.

## 2. Case Report

A 25-year-old male presented to the emergency department 2 months post-LSG with an unremarkable postoperative course. At presentation, he had a left upper quadrant abdominal pain associated with repeated vomiting, subjective fever, generalized body aches, and cough for two weeks. The cough was productive of greenish sputum with a tinge of blood and provoked by any oral intake. A review of other systems was unremarkable.

Physical exam shows the following: RP 103 BPM, BP 129/70, RR 18, SpO_2_ 99%, and temperature: 36.4°C. Abdomen examination shows soft and lax, mild generalized tenderness especially in epigastrium, normal bowel sounds. Chest examination shows no increased work of breathing, normal chest expansion, percussion note slight dullness in the left lower zone, chest sounds decreased breathe sounds in the left lower zone with bronchial breathing, and fine crackles.

Diagnostic testing shows the following: WBC: 8.5 × 10^3^/*μ*L (normal range 4‐10 × 10^3^/*μ*L), CRP: 6.7 mg/L (normal range 0-5 mg/L); blood and sputum culture were negative.

**Figure 1:**
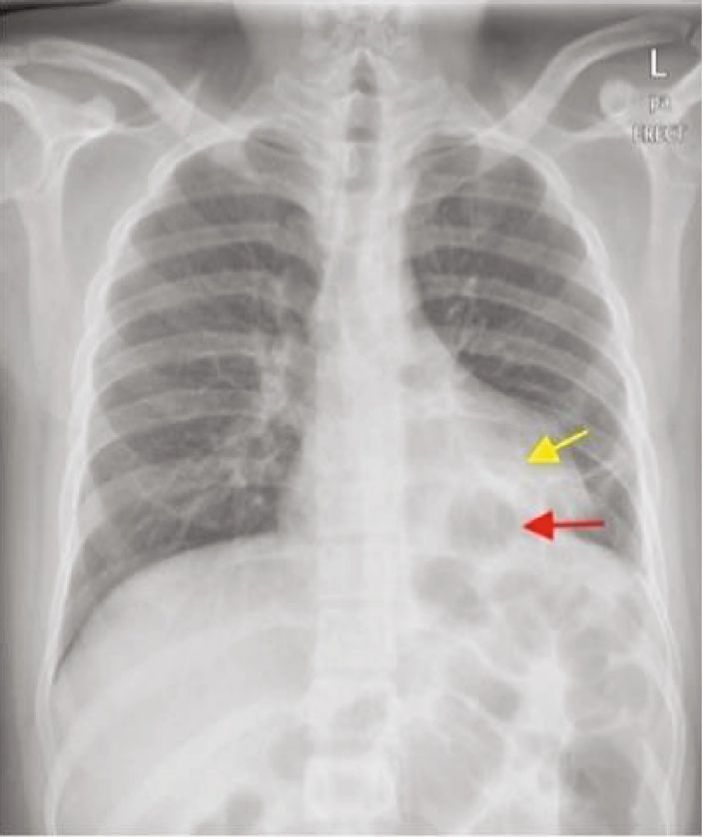
PA chest radiograph shows a left lower zone heterogeneous opacity silhouetting the left hemidiaphragm (yellow arrow) and a retrocardiac air lucency (red arrow).

**Figure 2:**
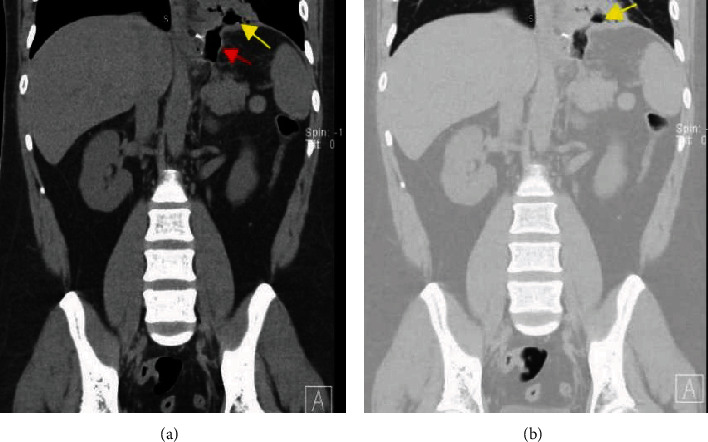
Coronal nonenhanced CT image of the lower chest and upper abdomen (soft tissue and lung window (a, b)) shows left lower lobe consolidation with cavitation (yellow arrow), communicating with the lumen of the gastric remnant (red arrow).

**Figure 3:**
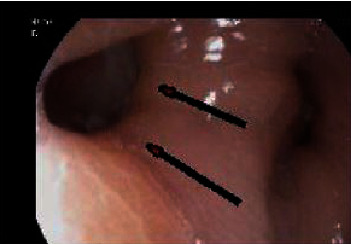
Esophagogastroduodenoscopy (EGD) was performed which showed a large fistula orifice just below GEJ (arrow).

Initial chest radiograph (Figure 1) showed a left lower lung zone heterogeneous opacity silhouetting the left hemidiaphragm and left retrocardiac air lucency. A follow-up CT scan of the abdomen (Figure 2) was done to rule out an intra-abdominal fluid collection (oral contrast was not given as the patient was intolerant of oral intake) and showed a left lower lobe consolidation with cavitation and tract connecting it with the gastric remnant lumen. The gastroenterology team was consulted, and esophagogastroduodenoscopy (EGD) confirmed the presence of a large fistula orifice just below the gastroesophageal junction (Figure 3).

**Figure 4:**
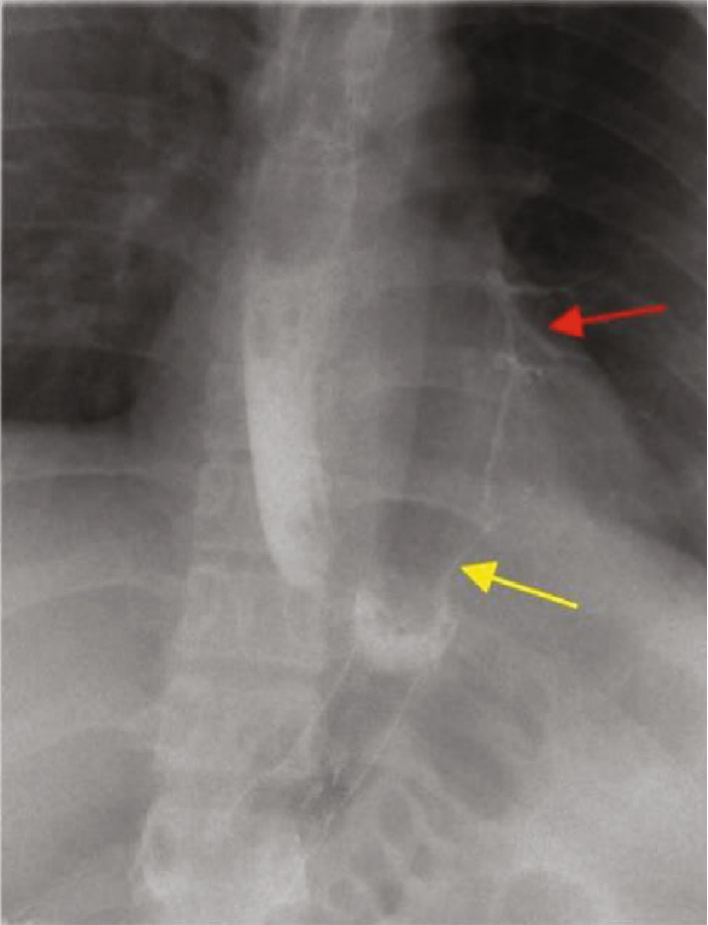
A single-contrast upper GI study performed with a water-soluble contrast shows contrast leak (yellow arrow) extending upwards from the gastric remnant into the left lung base with contrast opacification of the left lower lobe peripheral bronchial branches (red arrow) producing a gastrobronchial fistula.

For more anatomic delineation of gastrobronchial fistula, an upper GI study with water-soluble contrast (Figure 4) was performed which showed contrast leakage extending upwards from the gastric remnant into the left lung base with contrast opacification of the left lower lobe peripheral bronchial branches.

After confirmation and proper surgical planning of gastrobronchial fistula, bariatric surgery was intervened by the laparoscopic approach. The operative technique was resection of the fistula (found connected to GE junction) and gastrojejunal anastomosis. The patient had a successful postoperative recovery.

## 3. Discussion

Moeller and Carpenter first classified the causes of gastrobronchial fistula into five categories: (1) trauma, (2) esophageal or gastric surgery, (3) neoplasm, (4) gastric ulcer, and (5) subphrenic abscess [11]. Gastric fistula incidence following bariatric surgery has been estimated as 0.9–2.6%, reaching 10% in revision operations, with the angle of His being the most common location [12]. It has been suggested that ischemia in the gastric wall surrounding the staple line is more likely related to most cases of leak and subsequent fistula formation rather than staple line dehiscence [13]. The leaked secretion eventually drains in the subphrenic region resulting in abdominal sepsis and gastrocutaneous fistula, or it can form a gastrogastric fistula, the latter being less frequent. In rare circumstances, as in our case, the subphrenic abscess can cross the diaphragm and form a gastrobronchial fistula [14].

Distal stenosis following LSG may be the explanation of development of GBF, which decreases gastric emptying and thereby increases pressure in the stomach and directing the gastric contents into the fistula tract. This facilitates persistent communication between the stomach and the respiratory tract [11, 15].

The leak of gastric contents can lead to the development of a subphrenic abscess, which may spread above the diaphragm facilitated by lymphatic flow or by direct erosion through the diaphragm, leading to the development of a lung abscess that may end up opening into a bronchial tree [16]. The development of gastrobronchial fistula can also occur secondary to iatrogenic diaphragmatic injury after CT-guided drainage of the subphrenic abscess [17].

In our case, the postoperative period was unremarkable with no history of intervention, so we propose that the gastrobronchial fistula occurred secondary to a chronic silent microleak, which might be not significant to cause symptoms. That said, this possibly resulted in the spread of gastric contents into the left lower lung lobe creating a gastrobronchial fistula.

Clinically, patients present with symptoms related to the development of a subphrenic abscess (abdominal pain and fever), pulmonary symptoms (such as chronic productive cough, hemoptysis, and dyspnea), or even vomiting and expectoration of food particles and gastric contents [16, 18–20]. One characteristic clinical finding is that significant bouts of cough are caused by any food intake. Some authors have also described persistent hypercapnia despite high minute ventilation, which is due to increased physiologic dead space due to leakage of part of each breath into the stomach [19, 21]. In a recent multicenter retrospective study of 13 patients with gastrobronchial fistula following LGS, the mean duration before the appearance of symptoms was found to be 129 days [19].

Due to the very rare incidence of gastrobronchial fistula with limited published data in radiology literature, the diagnosis of gastrobronchial fistula is challenging to radiologists. CT is more likely to identify secondary signs, though less likely to identify the fistulous tract itself. Endoscopy does not diagnose GBF but can identify its internal opening, as seen in our case.

Despite the scarcity of evidence-based data about the gold standard diagnostic test for detection of GBF after LGS, a contrast study of the upper gastrointestinal tract is the widely accepted means of diagnosing a GBF [10, 15, 22, 23].

When the upper gastrointestinal study was considered in our case, there was a dilemma regarding which type of contrast material to choose. There are several types of contrast materials used for UGI series. Barium sulphate is most commonly used. However, due to the concerns of gastric leak, this agent was not used as it can lead to peritonitis. Water-soluble contrast materials (e.g., Omnipaque and Gastrografin), on the other hand, are safer in case of suspected leak because they are absorbed rapidly from the interstitial spaces and peritoneal cavity [24].

Another potential risk taken in consideration was pulmonary aspiration as the patient's clinical presentation of postprandial cough and vomiting raised suspicion of severe gastroesophageal reflux or fistula, which can result in aspiration to an uncertain extent. High osmolality contrast materials (e.g., Gastrografin) may, if introduced into the bronchial system due to aspiration or by fistula as in our case, induce severe bronchial irritation and life-threatening pulmonary edema. In our case, low osmolar iodinated contrast (Omnipaque 350) was used as it safe in case of suspected leak or aspiration [24].

The treatment of gastrobronchial fistulas should be tailored to the clinical state of the patient, and in the absence of sepsis, conservative management shall be tried [9]: management with wide-spectrum antibiotics, CT-guided percutaneous drainage of any subphrenic collection, endoscopic covered stent of gastric fistula, and nasojejunostomy feeding [10, 11]. If nonsurgical measures fail to treat GBF, surgical treatment with laparotomy and thoracotomy with the resection of the fistula and rarely total gastrectomy and lobectomy may be required [14, 18, 20, 25, 26].

## 4. Conclusion

Although the development of gastrobronchial fistula postlaparoscopic sleeve gastrectomy is rare, it is a serious and a difficult complication to manage. Post-LSG gastrobronchial fistula is not reported widely in radiologic literature; hence, confirming the diagnosis might be challenging; however, a contrast study of the upper GI tract is considered the ideal diagnostic test. Most of the post of the reported cases have evidence of postoperative gastric leak/subphrenic collection, in contrast to our case which has unremarkable postoperative recovery. Management of GBF should be individualized to each case, and it ranges from conservative and noninvasive to aggressive surgical approaches.

## Additional Points

*Institutional Approval*. This case report got institutional approval from Medical Research Center (MRC), Hamad Medical Corporation, Doha, Qatar. The approval letter number is MRC-04-20-412. The Medical Research Center has granted permission for this case report to be published on the condition that no patient-identifiable data (including patient name and photograph) are included.
